# Prostate brachytherapy seed migration to the right renal artery due to right‐to‐left shunting across a patent foramen ovale

**DOI:** 10.1002/iju5.12707

**Published:** 2024-03-07

**Authors:** Makoto Nakiri, Kosuke Ueda, Ryuji Hoshino, Naoki Ito, Hirofumi Kurose, Shoichiro Nohara, Koichiro Muraki, Chikayuki Hattori, Etsuyo Ogo, Tsukasa Igawa

**Affiliations:** ^1^ Department of Urology Kurume University School of Medicine Kurume Japan; ^2^ Division of Cardiovascular Medicine, Department of Internal Medicine Kurume University School of Medicine Kurume Japan; ^3^ Department of Radiology Kurume University School of Medicine Kurume Japan

**Keywords:** brachytherapy, foreign‐body migration, kidney, patent foramen ovale, prostate cancer

## Abstract

**Introduction:**

The seeds used in brachytherapy for prostate cancer may migrate through the surrounding venous plexus to other sites in the body, most commonly to the pulmonary vasculature.

**Case presentation:**

A 78‐year‐old Japanese man received iodine‐125 low‐dose‐rate prostate brachytherapy. Computed tomography revealed that one seed had migrated to the right kidney. No seed was observed in the ureter upon ureteroscopy. Transesophageal echocardiography confirmed a right‐to‐left shunt due to a patent foramen ovale, suggesting that the seed had migrated into the right renal artery. Three years after treatment, no recurrence of prostate cancer and no adverse events due to seed migration or due to the patent foramen ovale occurred.

**Conclusion:**

Arteriovenous malformations and a right‐to‐left shunt should be suspected if a brachytherapy seed has migrated to an artery of the systemic circulatory system.

**Abbreviations & Acronyms**
125I: iodine‐125
CT: computed tomography
LDB: low‐dose‐rate brachytherapy
PFO: patent foramen ovale
TEE: transesophageal echocardiography

**Keynote message**

This is the first report demonstrating that a right‐to‐left shunt due to a patent foramen ovale caused migration of a prostate brachytherapy seed to the right renal artery. We believe that the seed migrated into the pelvic vein, reached the right atrium, and flowed into the systemic circulation directly through the patent foramen ovale without passing through the pulmonary circulation, ending up in the right renal artery. If a brachytherapy seed migrates into an artery of the systemic circulation, a pulmonary arteriovenous malformation or right‐to‐left cardiac shunt should be suspected. A diagnosis of a patent foramen ovale and early therapeutic intervention may reduce the risk of secondary strokes.

## Background

Seed migration is a common event that occurs after 125I LDB for prostate cancer. Seed migration to the pulmonary vasculature has most frequently been reported. The seed less commonly migrates to the abdomen and pelvis, coronary artery, right ventricle, and kidneys.^1^, ^2^, ^3^, ^4^, ^5^, ^6^, ^7^ The prostate is surrounded by a well‐developed venous plexus, and the seed is implanted near or within the vein.^1^ Thus, seed migration is thought to be caused by the seeds entering the vessel and circulating through the blood.^8^ We report a rare case in which a right‐to‐left shunt due to a PFO caused seed migration to the right renal artery after LDB for prostate cancer.

## Case presentation

**Fig. 1:**
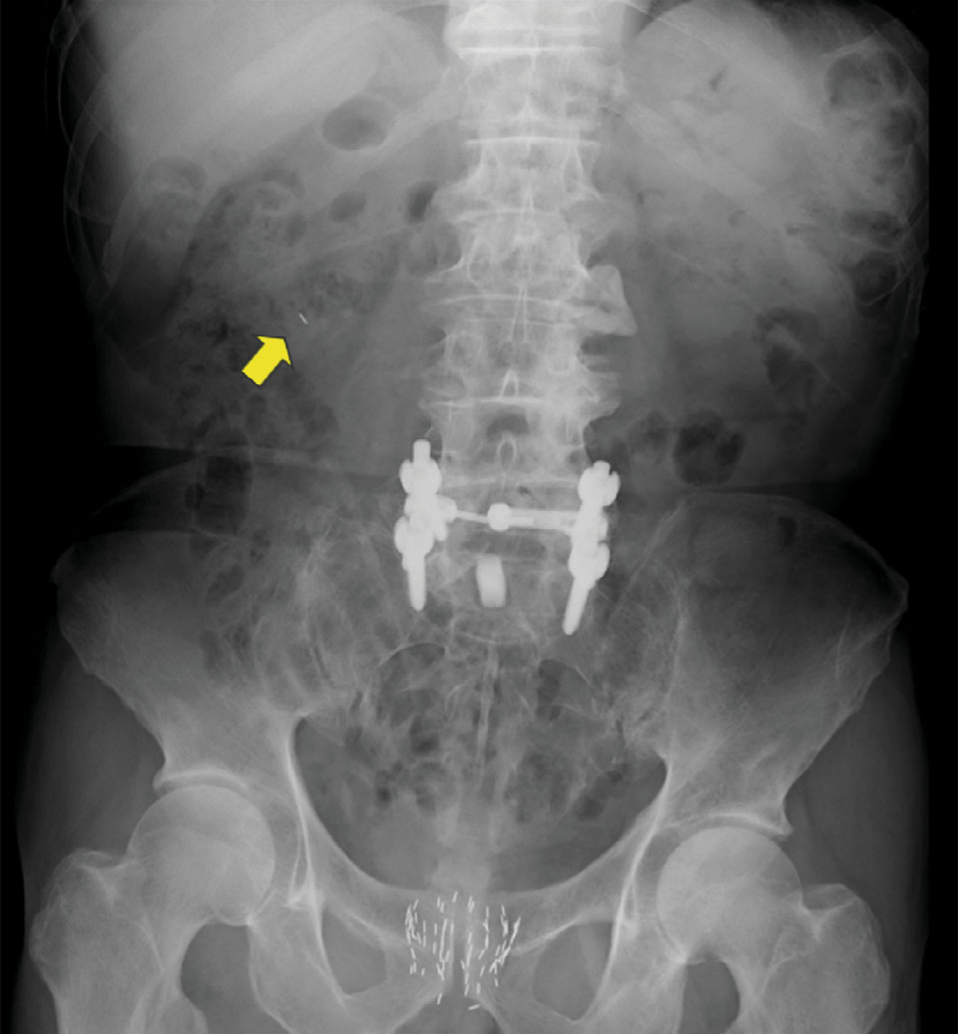
Pelvic radiograph revealing that a seed (yellow arrow) had migrated to the right side of the patient's middle abdomen.

**Fig. 2:**
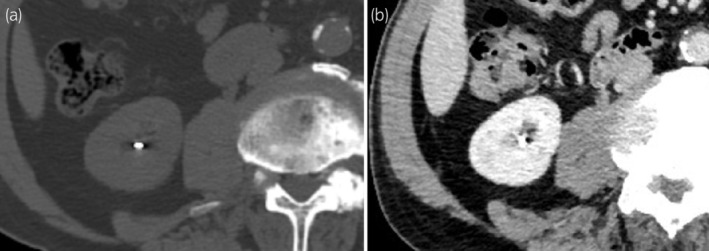
CT (a) immediately after and (b) 1 year after LDB, revealing that the seed had migrated to the right kidney.

**Fig. 3:**
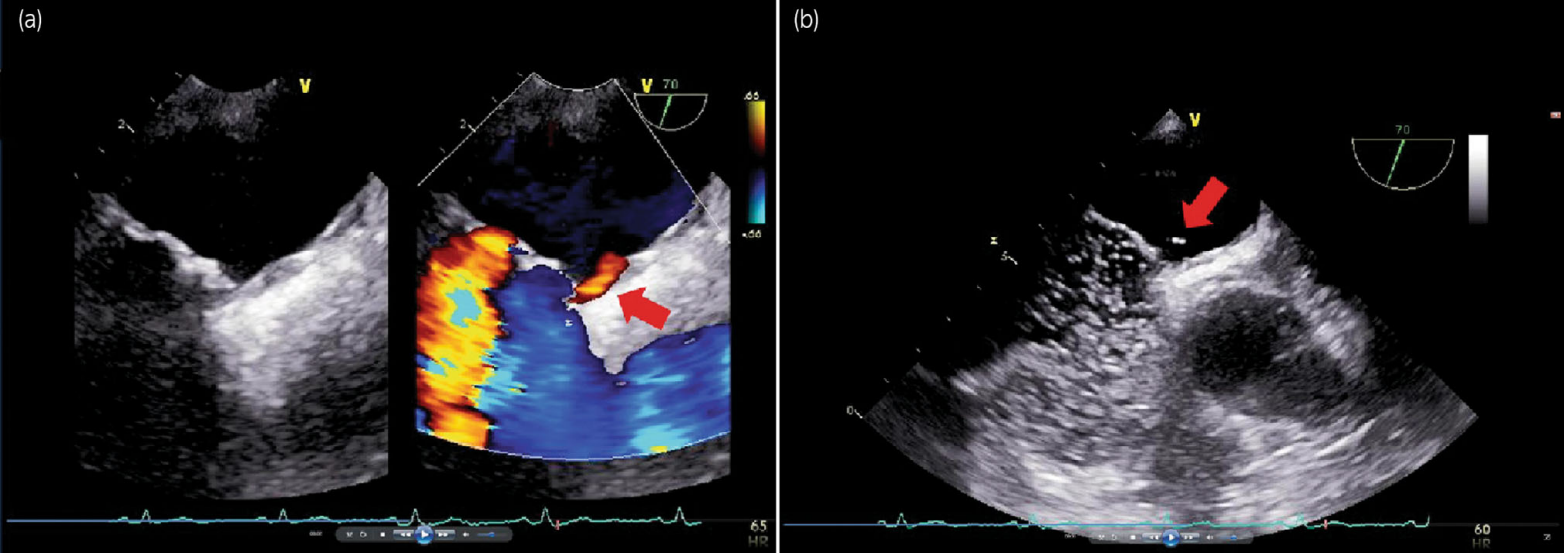
Shunt flow (a) and microbubbles (b) from the right atrium to the left atrium, observed after the Valsalva maneuver was released using TEE.

A 78‐year‐old Japanese man with no history of stroke was diagnosed with localized, intermediate‐risk prostate cancer (cT2aN0M0, Gleason score: 3 + 4 = 7, initial prostate‐specific antigen concentration: 4.88 ng/mL). 125I‐LDB (prescribed dose: 145 Gy) was performed. Seventy‐six seeds were placed without complications, one of which migrated into the patient's pelvis. The next day, a follow‐up pelvic radiograph indicated that seed had migrated to the right side of the middle abdomen (Fig. 1). CT revealed that the seed had migrated to the right kidney (Fig. 2). Although the exact location of the seed was difficult to determine, we suspected that it was in the urinary tract or renal vasculature. As adverse events due to the seed migration were considered unlikely, we monitored the patient without intervention. One year after LDB, contrast‐enhanced CT revealed that the seed had remained in the same area (Fig. 2). The patient had not had an inflammatory response or subjective symptoms. No seed was observed in the ureter upon ureteroscopy. We suspected the presence of a right‐to‐left shunt causing the seed to migrate into an artery via the systemic circulation. TEE confirmed a right‐to‐left cardiac shunt due to a PFO (Fig. 3). These results suggested that the seed had migrated into the right renal artery. Three years after treatment, no recurrence of prostate cancer and no adverse events due to seed migration or due to the PFO occurred.

## Discussion

LDB is an effective treatment for localized prostate cancer.^9^, ^10^ The radioactive seeds used in LDB are cylindrical, 4.5‐mm long, and 800 μm in diameter.^5^ They are placed at the periphery of the prostate, the most common area for prostate cancer. The surrounding blood vessels are large enough to accommodate hematogenous migration of the seeds to other sites.^1^ In one study, one or more seeds were identified upon chest radiography in 55% of patients who underwent prostate brachytherapy.^3^ Therefore, patients undergoing brachytherapy must undergo radiography. Even so, migration to the lungs may be missed in 10–15% of cases when chest radiographs alone are used to confirm migration.^11^ CT is reportedly very useful in detecting migrated seeds in the chest but may cause overexposure, as additional CT is needed if the seed causes an adverse event.^12^ Although rare cases of physical embolization of the seed and adverse events due to radiation have been reported,^5^ the migrated seed normally does not require intervention owing to its small size and half‐life of 59.4 days.^5^, ^6^, ^7^ Although the probability of adverse events due to seed migration is very low, the seed migration site should be carefully monitored for such events. Previous reports of seed migration to the lungs, pelvis, heart, mediastinum, kidney, inguinal canal, liver, urinary tract, and sacrum after brachytherapy have been published. Most cases are asymptomatic. However, various adverse events caused by seed migration have been reported. Nguyen *et al*.^6^ reported cases of back pain and asymptomatic gross hematuria due to seed migration into the renal artery. In addition, Chen *et al*.^13^ reported a case of stone formation due to seed migration into the bladder after brachytherapy. As determining whether the seed migrated to the renal vasculature or urinary tract is challenging when using only contrast‐enhanced CT, we performed ureteroscopy in this case for confirmation. We considered the possibility of excision if the seed had migrated into the urinary tract.

Migration of a single seed does not affect the dose covering 90% of the prostate volume (D90), but migration of two or more seeds reportedly decreases the D90 (Gy) value and the percentage of the prostate receiving 100% of the prescribed dose compared with no seed migration.^8^ However, the differences in radiation dose owing to migrated seeds are reportedly very small and have little impact on treatment outcomes.^12^

Seed migration into the coronary arteries or the systemic circulation may be caused by a right‐to‐left atrial or ventricular shunt.^14^ In this case, as the seed had migrated into the right renal artery, we assumed that the seed had migrated through the systemic circulation without passing through the pulmonary circulation, and thus a right‐to‐left shunt due to a PFO was suspected and confirmed. A PFO is a valve in the interatrial septum that allows the shunting of blood or thrombotic material between the atria. Options for the diagnosis of a PFO include transthoracic echocardiography, TEE, and transcranial Doppler ultrasonography. In a pooled analysis of autopsy studies, the mean prevalence of PFOs was 26% (range: 17–35%), with no pathologic association in the vast majority of cases.^15^ Most people with a PFO remain asymptomatic and do not require treatment. However, PFOs can cause a paradoxical thromboembolism to form in the veins and move into the systemic circulation. In a recent study of 61 patients, a PFO was discovered in 45% of those with cryptogenic stroke.^16^ Treatment options for secondary stroke prevention in the presence of a PFO include antiplatelet therapy, anticoagulation therapy, and percutaneous device closure.^17^ Rare cases of seed migration to the coronary artery, right ventricle, and right renal artery have been reported^5^, ^6^, ^7^; however, to our knowledge, this is the first report demonstrating that a right‐to‐left shunt due to a PFO might have caused seed migration to the right renal artery, which strongly supports the hypothesis of previous report.^6^ If the seed migrates into an artery of the circulatory system, a pulmonary arteriovenous malformation or right‐to‐left cardiac shunt should be suspected. PFO closure is recommended for secondary prevention of cryptogenic stroke.^18^, ^19^ This patient did not undergo PFO closure as he had no history of cerebral infarction. However, considering that the seeds entered his systemic circulation, he has to be carefully monitored for the occurrence of paradoxical cerebral embolism through the PFO in the future. Diagnosis of cryptogenic stroke can be difficult in clinical practice, and an awareness of the history of arterial embolism through a PFO is important as it may promote the early and appropriate diagnosis of the disease.

In conclusion, arteriovenous malformations and a right‐to‐left shunt should be suspected if a brachytherapy seed has migrated to an artery of the systemic circulatory system.

## Author contributions

Makoto Nakiri: Conceptualization; data curation; investigation; methodology; project administration; resources; validation; writing – original draft; writing – review and editing. Kosuke Ueda: Project administration; writing – original draft; writing – review and editing. Ryuji Hoshino: Conceptualization; data curation; methodology; project administration; writing – original draft; writing – review and editing. Naoki Ito: Data curation. Hirofumi Kurose: Data curation. Shoichiro Nohara: Data curation; project administration. Koichiro Muraki: Conceptualization; investigation; resources. Chikayuki Hattori: Methodology; resources. Etsuyo Ogo: Conceptualization; supervision. Tsukasa Igawa: Conceptualization; project administration; supervision; writing – review and editing.

## Conflict of interest

The authors declare no conflict of interest.

## Approval of the research protocol by an Institutional Reviewer Board

Not applicable.

## Informed consent

The patient provided written informed consent for all treatments and publication of the report.

## Registry and the Registration No. of the study/trial

Not applicable.

## Data Availability

The data that support the findings of this study are available from the corresponding author, M.N., upon reasonable request.
